# What About Compressing the Oesophagus with an Ultrasound Probe for a Modified Sellick Maneuver? 

**DOI:** 10.5152/TJAR.2021.1427

**Published:** 2022-02-01

**Authors:** Onat Bermede, Başak Ceyda Meço, Volkan Baytaş, Olcay Dilken, Çiğdem Yıldırım Güçlü, Süheyla Karadağ Erkoç, Zekeriyya Alanoğlu, Neslihan Alkış

**Affiliations:** 1 Department of Anaesthesiology and Intensive Care, Ankara University School of Medicine, Ankara, Turkey; 2 Department of Intensive Care, Istanbul University-Cerrahpaşa School of Medicine, Istanbul, Turkey; 3 Department of Anaesthesiology, Washington University School of Medicine in St. Louis, Saint Louis, USA

**Keywords:** Airway ultrasound, paralaryngeal pressure, rapid sequence induction

## Abstract

**Objective:**

Debates continue about the cricoid pressure, which has been used for many years to prevent gastric aspiration during intubation. Using ultrasound, the effects of this maneuver and alternatives like paralaryngeal pressure are revealed. The aim of this observational study was to determine the effect of paralaryngeal pressure with an ultrasound probe on the oesophageal diameter in patients with different body mass indexes and neck circumferences.

**Methods:**

After measuring the neck circumference at the level of the cricoid cartilage, the oesophagus was visualized by ultrasonography. Compression was applied medially at a 45° angle toward the vertebral column by the ultrasound probe and oesophageal anteroposterior outer diameters were measured. Correlations between body mass index, neck circumference, oesophageal diameter, and oesophageal diameter change ratio were evaluated with Pearson’s *r* value.

**Results:**

One hundred ten volunteers (52 women and 58 men) with mean age 33.7 ± 8.02 years and mean body mass index 25.6 ± 4.65 kg m^−2^ were recruited. The oesophagus was located 78.18% partially to the left, 4.54% completely to the left, 1.81% to the right of the cricoid ring. In 15.45%, oesophagus could not be displayed. The mean diameter of the oesophagus was 7.6 ± 1.1 mm before pressure and 5.6 ± 0.09 mm after pressure (*P* < .001). There was no significant correlation between diameter change percentage and body mass index (*r* = −0.22; *P* > .05). However, weak correlation was found between diameter change percentage and neck circumference (*r* = −0.33; *P* = .016).

**Conclusions:**

Paralaryngeal pressure with an ultrasound probe has the potential to occlude the oesophagus and may be effective in all patient groups.

**Main Points**

- Contrary to popular belief, the oesophagus lies down most often to the left of the trachea, then behind and to the right, respectively.
- Paralaryngeal pressure can be applied more standardized by visualizing by ultrasound and decreases the diameter of oesophagus.
- Regardless of body mass index and neck circumference, a significant narrowing of oesophagus occured with paralaryngeal compression.

## Introduction

Cricoid pressure (CP), known as Sellick’s maneuver, which is one of the important components of rapid sequence anaesthesia induction, has been used for years to seal the oesophagus between the cricoid cartilage and the body of the fifth cervical vertebra to prevent aspiration of gastric contents.^1^ However, its effectiveness in preventing aspiration is still controversial.^2,3^

Computed tomography (CT) and magnetic resonance imaging (MRI) studies have shown that the oesophagus is located lateral to the cricoid ring in more than 50% of subjects and this rate rises to 90% with CP.^4^ In addition, it has been reported that adequate closure of the oesophagus cannot be achieved with CP and the main closure is mostly in the hypopharynx.^5,6^

Due to the lateral location of the oesophagus, paralaryngeal pressure (PLP) is suggested as an alternative to CP. In terms of functional effect, it has been shown by ultrasonography (which can be easily reached in the operating theater) that PLP decreased the anteroposterior (AP) diameter of the upper oesophagus significantly in conscious volunteers.^7^

In this context, the primary aim of this study was to investigate the effect of PLP with the ultrasound probe on the oesophageal AP diameter in patients with different body mass indexes (BMI) and neck circumferences. During evaluating the neck anatomy by ultrasound, we also aimed to assess the correlation between anthropometric measurements (BMI and neck circumference) and the oesophageal diameter.

## Methods

This prospective observational study was performed in accordance with the Declaration of Helsinki. After ethical approval and informed consent, 110 adult volunteers, who were the staff of operating theater, enrolled in the study. Demographic data, height, and weight of patients were recorded. 

**Figure 1.**
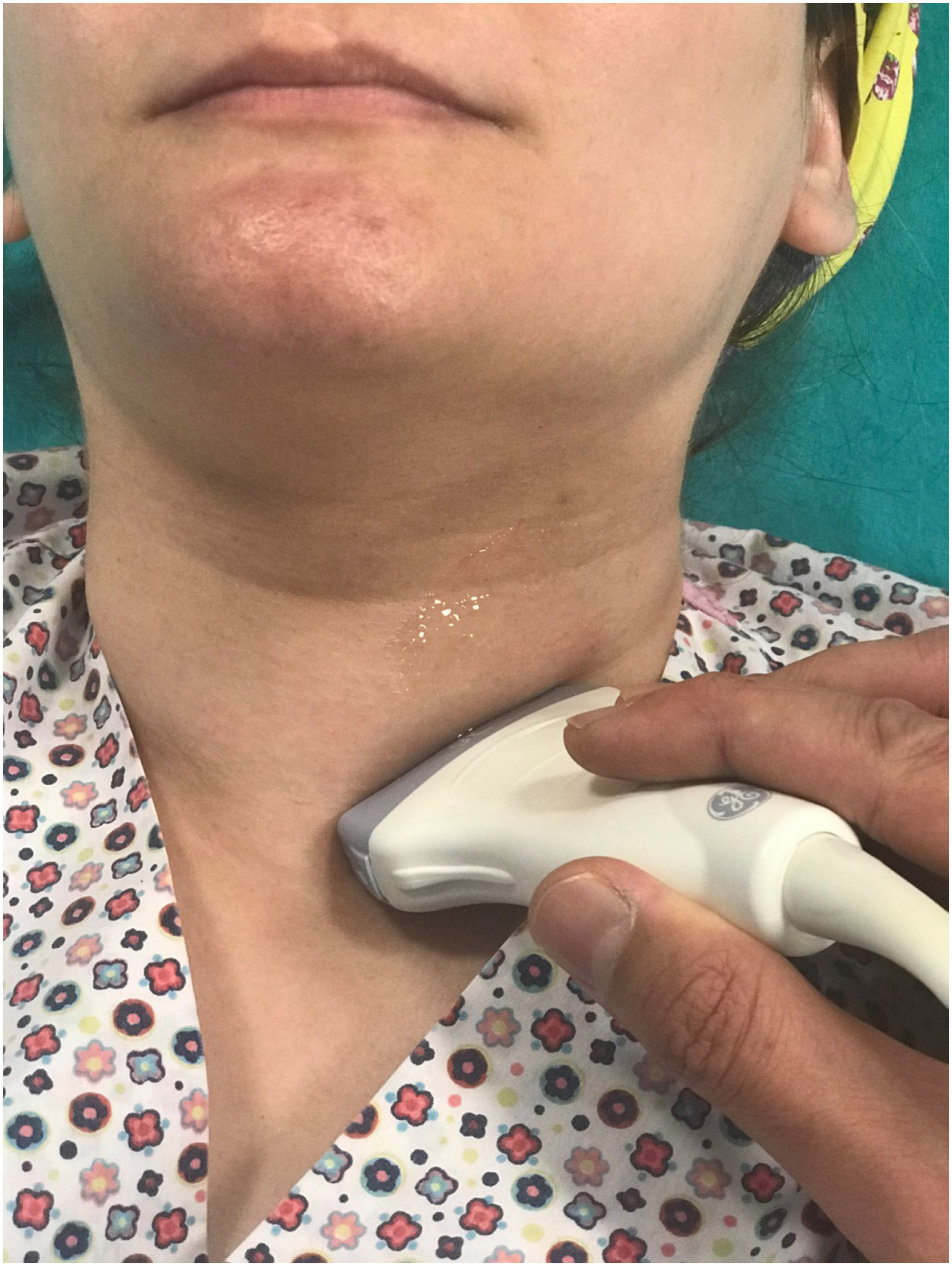
Placement of the ultrasound probe for paralaryngeal pressure.

Afterward, the neck circumference was measured at the level of the cricoid cartilage while volunteers were in the supine position with the head extended. Prior to ultrasound evaluation, all participants were monitored with three-lead electrocardiogram and pulse oximetry. In all volunteers, the oesophagus was visualized in the paralaryngeal area by ultrasonography by the same physician using GE Logiq E with a 3.3-10 MHz linear probe with a footprint length of 53 mm (Figure 1). 

The images were recorded without measurement. By the same physician, compression was applied medially at a 45° angle toward the vertebral column by the ultrasound probe and the images were recorded again. For the 30 N compression force standardization, at least 50 compressions of a weighing scale were performed before the study. Competence in the application of the desired PLP was assured by 20 consecutive successful applications of a 30 N force (within a range of 2 N). 

Oesophageal AP outer diameter measurements on all recorded images were performed by another independent and experienced physician, who was blinded to the study. The AP measurement was performed in the middle of the transverse diameter, between the external layers of the walls of the oesophagus.

Correlations among BMI, neck circumference, oesophageal diameter, and oesophageal diameter change ratio were evaluated.

## Statistical Analysis

An a priori power analysis was conducted using G*Power (Universitat Düsseldorf, Germany) to test the difference between 2 dependent group means using a two-tailed test, a medium effect size (*d* = 0.50), and an alpha of 0.05. Results showed that a total sample of 54 participants was required to achieve a power of 0.95. This number was inflated to 60 to account for possible losses in image acquisition. Continuous values are shown as mean (standard deviation [SD]). The distribution of the groups was evaluated with the Kolmogorov–Smirnov test. The correlation between normally distributed parameters was evaluated with Pearson's *r* value. *P* < .05 was considered statistically significant.

## Results

A total of 110 volunteers, including 52 women and 58 men, were recruited. The mean age was 33.7 ± 8.02 years, and the mean BMI was 25.6 ± 4.65 kg m^−2^. 

**Figure 2.**
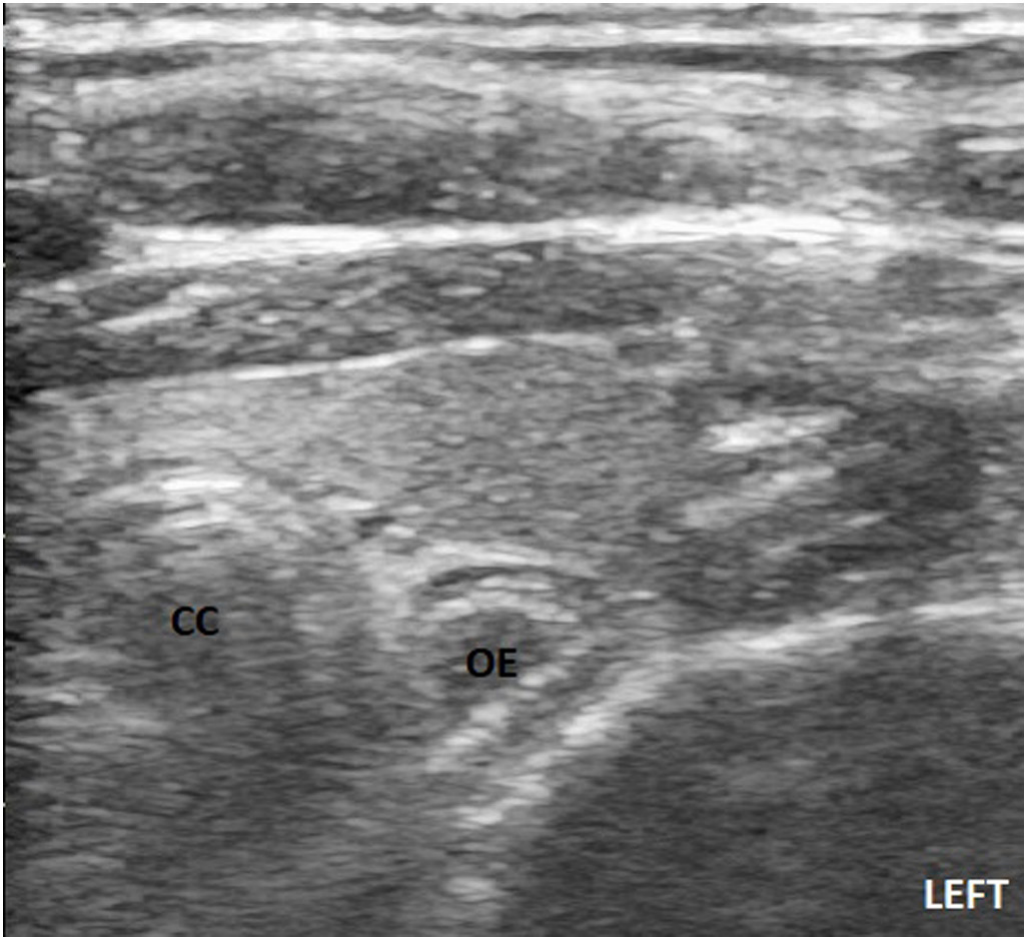
Ultrasound image of oesophagus before paralaryngeal pressure.

Before PLP, 78.18% (86) had esophagi positioned partially to the left of the cricoid ring, 4.54% (5) completely to the left of the cricoid ring, 1.81% (2) right of the cricoid ring, and in 15.45% (17) oesophagus was not visualized due to posterior placement during the ultrasound examination (Figure 2). 

**Figure 3.**
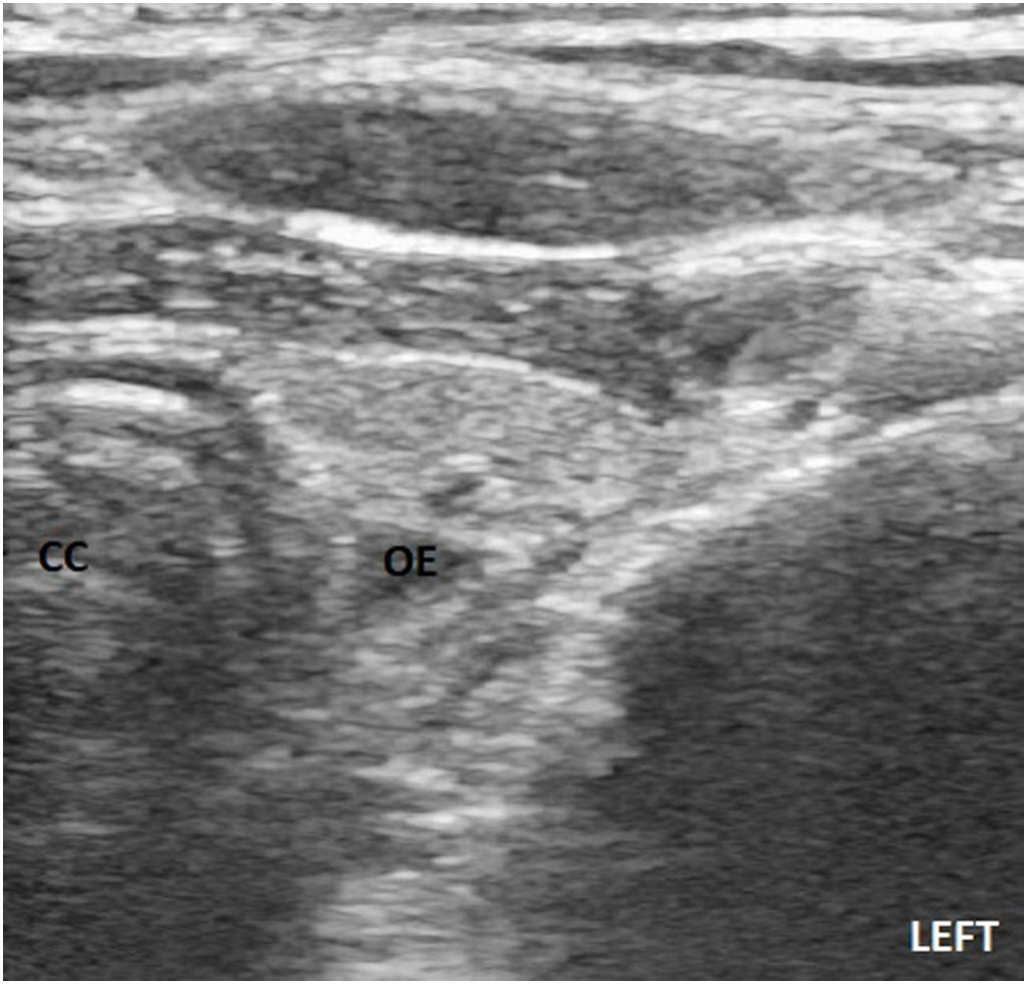
Ultrasound image of oesophagus after paralaryngeal pressure.

When PLP was applied using the ultrasound transducer, the oesophagus was visualized completely to the left in 58.18% (64), partially to the left in 30.90% (34) and partially to the right in %1.81 (2). In 9.09% (10) of the volunteer’s oesophagus was not visualized after compression (Figure 3) (Table 1). 

Diameter measurements could not be made in 17 volunteers with the oesophagus located behind the trachea before the PLP. In addition, 3 volunteers, whose oesophagus was behind the trachea after compression, although it was lateral at the first evaluation excluded.

The mean diameter of the outer oesophagus was 7.6 ± 1.1 mm in the neutral position and 5.6 ± 0.09 mm after pressure with the transducer (*P* < .001). 

**Figure 4.**
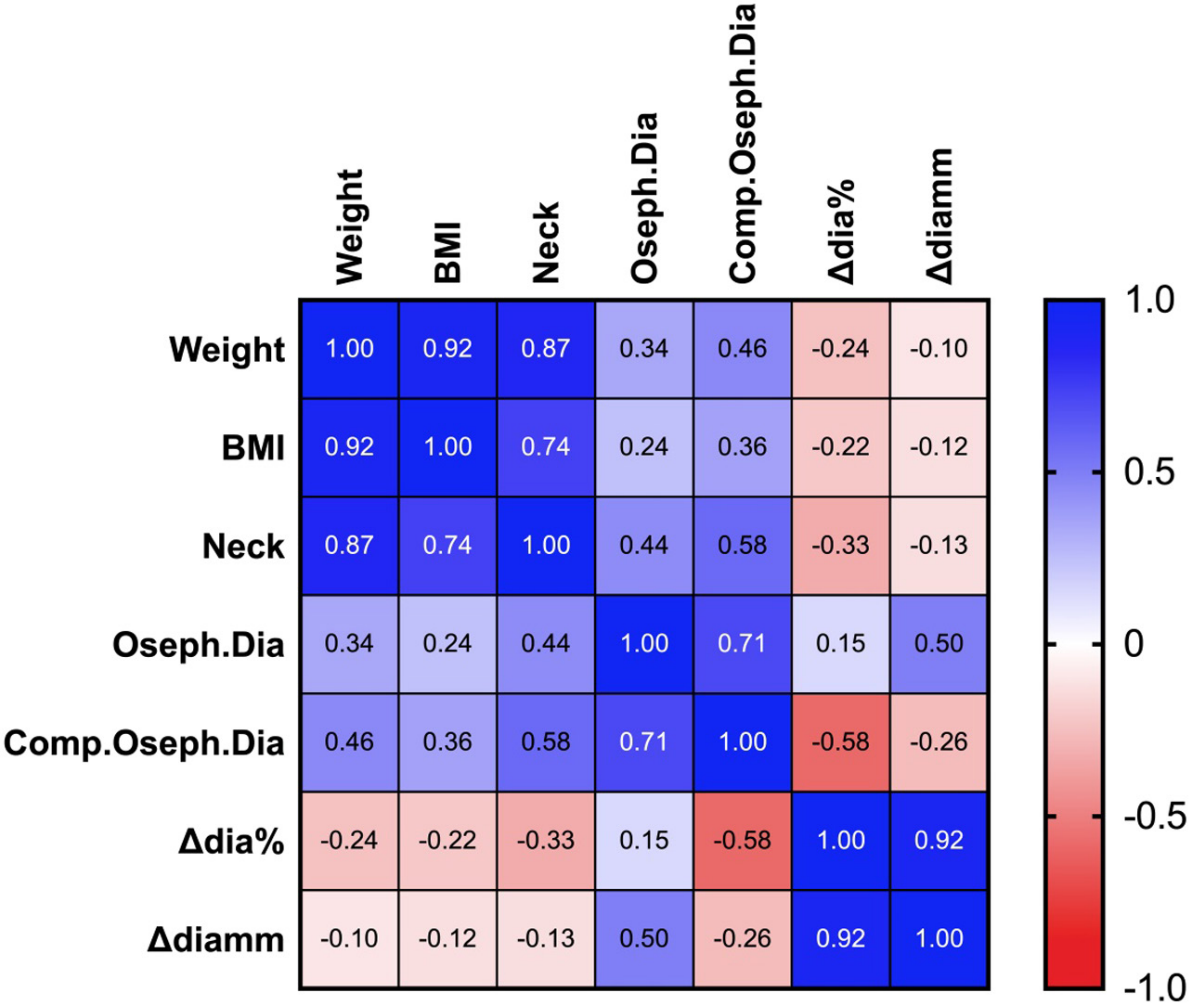
Pearson’s r correlation between the parameters.

There was a strong positive correlation between BMI and neck circumference (*r* = 0.74, *P* < .001). Any correlation between oesophageal diameter and BMI cannot be found (*r* = 0.24; *P* > .05). The correlation between neck circumference and oesophageal diameter was weak (*r* = 0.44; *P* < .001). There was no significant correlation between diameter change percentage and BMI (*r* = −0.22; *P* > .05). However weak correlation was found between diameter change percentage and neck circumference (*r* = −0.33; *P* = .016) (Figure 4).

## Discussion

This study demonstrated that, in all patients regardless of BMI and neck circumference, a significant narrowing in the oesophagus occurred with paralaryngeal compression. The neck circumference was negatively correlated with the diameter change percentage throughout PLP via ultrasonography. As expected, a strong positive correlation between BMI and neck circumference in adult volunteers was demonstrated. However, there was no correlation between BMI and any oesophageal measurement for this study.

Rapid sequence anaesthesia induction still maintains its importance. Standardization of different components of this application (neuromuscular blockade, opioid usage, and CP) is very important in terms of providing common approaches and will provide convenience for practitioners. Ultrasonography, which is used frequently in the operating room, may also be a part of this technique. In this article, the objective effect of a different application of ultrasonography in anaesthesia practice is shown.

It has been demonstrated with the previous CT and MRI studies that the oesophagus usually lays partially or completely next to the trachea.^8^ Afterward, it is revealed that this relationship was dynamic and the location of the oesophagus changes with pressure. Even with CP, the rate of positioning completely laterally is up to 70%. Tsung et al.^9^ determined by ultrasound that this positioning is up to 80% to the left of the trachea, to a lesser extent in the posterior of the trachea. Also, Kei et al.^10^ reported that without CP, the oesophagus laid 20% directly behind the trachea, 60% partially behind the trachea, and 20% completely lateral to the trachea. Similarly, in our study, it was observed that the oesophagus laid 78.18% partially left, 4.54% completely left, 15.45% posterior, and 1.81% partially right to the trachea without any pressure. 

This anatomy and dynamic relationship led to the idea that adequate closure of the oesophagus cannot be achieved with CP. Boet et al.^5^ stated in an MRI study that the effective application of CP resulted in incomplete occlusion of the oesophageal lumen. Inadequate oesophageal occlusion with CP conceived the emergence of the concept of PLP. Andruszkiewicz et al.^6^ found that the PLP creates a much more significant narrowing of the oesophagus than CP in healthy volunteers. This study appears to be the first to use ultrasound to evaluate oesophageal occlusion with CP. They reported that despite CP does not reduce the anteroposterior diameter of the oesophagus; PLP of 30 N by an ultrasound probe with a footprint length of 23 mm decreases this diameter and has the potential to occlude the upper oesophagus. Since the value of 30 N was taken as a cut-off for both cricoid and paralaryngeal compression in previous studies, we aimed to apply this pressure in patients with different demographic characteristics.^11^ These findings led us to apply PLP with the ultrasound probe. However, differently, we worked with the standard linear probe with a footprint length of 53 mm, which would be more acquirable in all centers. This standard probe was placed obliquely because it is oversize in the short axis placement and does not create an effective compression in the long axis placement. As in similar studies, we detected a significant reduction in oesophageal anteroposterior diameter with PLP.

To our knowledge, the effects of PLP on different body sizes have not been studied before. The frequency of obesity, one of the most important problems of today, is constantly increasing. We aimed to reveal the effect of PLP, which is still controversial in the literature, on volunteers with different physical characteristics.

A strong positive correlation was found between BMI and neck circumference. Nevertheless, any relation between BMI and oesophageal diameter was not found. More importantly, there was not any correlation between BMI and diameter change percentage. These findings may prove that PLP can be effective in patients regardless of BMI. Also, negative weak correlation between neck circumference and diameter change percentage shows that physical status does not interfere with this application. 

In contrast to these views of the current study, Rice et al.^7^ ­emphasized that the CP acts by closing the post-cricoid hypopharynx, not the oesophagus. Afterward, Zeidan et al.^12^ demonstrated using video laryngoscope that the main effect of CP is at the level of the oesophageal entrance at the hypopharynx level. They published that CP administration closed the oesophageal entrance and prevented gastric tube placement in all patients under general anaesthesia. In a recent study comparing the effects of CP and PLP, Kim et al.^13^ reported that both manipulation decrease the diameter of the upper oesophageal entrance but the occlusion of the oesophageal entrance is achieved more frequently with CP than PLP during direct laryngoscopy. From these studies, the idea of using both maneuvers in combination may arise in patients with high aspiration risk. 

Cricoid pressure and PLP will often be needed under emergency conditions. Although it has become easier to access ultrasound in emergency rooms or operating theaters lately, it may not be possible to find a suitable ultrasound probe. However, in correlation with previous studies, it has been found that the oesophagus often lies on the left side of the larynx. Even if there is no ultrasound probe in patients with high aspiration risk, in addition to cricoid compression, paralaryngeal pressure applied to the left side with the finger can reduce the oesophageal diameter and decrease the risk of aspiration.

This study has several limitations. First, the volunteers who participated in the study were mostly young people with mean age of 33.7 ± 8.02 years, without the concomitant disease. This sample will not adequately reflect the population of patients in the operating room, especially in emergency situations. In order to minimize this probability, more volunteers were enrolled in the study than in similar studies.

Secondly, an investigator performed at least 50 compressions of a weighing scale for the 30 N compression force standardization before the study, and competence in the application of the desired PLP was assured by 20 consecutive successful applications of a 30 N force (within a range of 2 N). However, the paralaryngeal force applied continuously could not be monitored. Possible errors in the amount of applied force cannot be excluded.

Third, the evaluations were made in volunteers who were not under general anaesthesia and muscle relaxant effect. Therefore, performing the same measurements after induction of anaesthesia can lead to some differences due to the dynamic relationship between trachea and oesophagus.

## Conclusion

Paralaryngeal pressure decreases the diameter of the oesophagus and has the potential to occlude the oesophagus. This pressure can be applied more standardized by visualizing using the ultrasound probe. Although there is a relationship between BMI and neck circumference, this may not be reflected in the oesophageal diameter change percentage, which may lead to the idea that PLP by an ultrasound probe or a finger may be effective in all patient groups. In patients with a very high risk of aspiration, both maneuvers, CP and PLP, can be performed together.

## Figures and Tables

**Table 1.** Position of Oesophagus Relative to the Trachea Before and After PLP

Position of Oesophagus	Before PLP n (%)	After PLP n (%)
Partially behind trachea (left)	86 (78.18)	34 (30.90)
Completely lateral to trachea (left)	5 (4.54)	64 (58.18)
Partially behind trachea (right)	2 (1.81)	2 (1.81)
Directly behind trachea	17 (15.45)	10 (9.09)

PLP, paralaryngeal pressure.
